# Supplementary material for: Robotic Living Donor Right Hepatectomy: A Systematic Review and Meta-Analysis
Source: J Clin Med. 2022 May 5;11(9):2603. doi: 10.3390/jcm11092603 (PMC9103024; doi:10.3390/jcm11092603)
Supplement: Supplementary file 1 [file jcm-11-02603-s001.zip › jcm-1672349-supplementary.pdf]

Supplemental Digital Content

Supplemental Table S1. Risk of Bias of Included Studies

Study	Score								
**CLARITY Tool to Assess Risk of Bias in Cohort Studies**	1. Was selection of exposed and non-exposed cohorts drawn from the same population?	2. Can we be confident in the assessment of exposure?	3. Can we be confident that the outcome of interest was not present at start of study?	4. Did the study match exposed and unexposed for all variables that are associated with the outcome of interest or did the statistical analysis adjust for these prognostic variables?	5. Can we be confident in the assessment of the presence or absence of prognostic factors?	6. Can we be confident in the assessment of outcome?	7. Was the follow up of cohorts adequate?	8. Were co-interventions similar between groups?	Overall
**Rho et. al, 2020**	Unclear	Low	Low	Low	Unclear	Low	Low	Low	Unclear
**Broering et. al, 2020**	Low	Low	Unclear	Low	Unclear	Low	Low	Low	Unclear
**Binoj et. al, 2020**	Unclear	Unclear	Unclear	Unclear	Unclear	Unclear	Unclear	Unclear	Unclear
**Chen et. al, 2016**	Low	Unclear	Low	Unclear	Unclear	Low	Unclear	Low	Unclear

Supplemental Table S2. Donor preoperative and postoperative characteristics

Author/Year	Intervention	Population number	Age (years, mean ±SD.)	Female (%)	BMI (mean ±SD.)	Total liver volume (ml, mean ±SD.)	Graft volume (ml, mean ±SD.)	Conversion to open (%)	Warm ischemia time (min ±S.D.)	Mortality (%)
**Rho et. al, 2020**	Robotic	52	28.6 (8.7)	50.0	22.4 (2.1)	1178.2 (172.4)	718.9 (104.3)	2.0	15.7 (6.4)	NR
	Laparoscopic	118	36.9 (12.1)	37.3	23.3 (2.5)	1253.4 (216.2)	785.1 (144.2)	NR	NA	NR
	Open	62	28.7 (8.3)	37.1	22.1 (2.4)	1175.0 (181.1)	731.3 (124.2)	NA	NA	NR
**Broering et. al, 2020**	Robotic	35	28.6 (6.0)	37.1	23.4 (2.9)	NR	701.0 (148.0)	0.0	8.4 (2.3)	0.0
	Open	70	NR	34.3	NR	NR	701.0 (133.0)	NA	2.3 (1.2)	0.0
**Binoj et. al, 2020**	Robotic	51	37.9 (10.5)	66.7	NR	NR	690.6 (131.6)	NR	15.4 (4.4)	NR
	Open	62	40.6 (10.0)	74.1	NR	NR	653.5 (144.2)	NA	7.9 (3.6)	NR
**Chen et. al, 2016**	Robotic	13	NR	69.2	21.9 (2.9)	1147.2 (148.2)	617.7 (140.5)	0.0	9.5 (8.0 – 15.0)*	NR
	Open	54	NR	55.6	22.7 (3.5)	1231.9 (292.6)	632.4 (120.9)	NA	NR	NR
**Total**	Robotic	151	32.0 (4.6)	54.3	22.7 (0.7)	1172.0 (18.4)	696.5 (38.6)	0.1	13.3 (1.6)	0.0
	Laparoscopic	118	36.9 (12.1)	37.3	23.3 (2.5)	1253.4 (216.2)	785.1 (144.2)	NR	NR	NR
	Open	248	34.7 (5.9)	49.6	22.4 (0.3)	1201.5 (27.6)	683.4 (38.1)	NA	4.9 (1.2)	0.0

NR: Not Reported; NA: Not Applicable. * range

Supplemental Table S3. Patient inclusion and selection criteria for the robotic approach

Author/Year	Robotic transplantation (n)
Rho et. al, 2020	Inclusion criteria were graft volume (initial <800 g), the presence of an inferior right hepatic vein, and bile duct anatomy and portal vein anatomy. Around the 10th case, the authors expanded their criteria to donors with bile duct trifurcation and portal vein trifurcation.
Broering et. al, 2020	Inclusion criteria were normal anatomy or multiple bile ducts, double arterial inflow, and portal trifurcation, remnant more than 30-35%, and graft-to-recipient weight ratio over 1%,
Binoj et. al, 2020	Inclusion criteria were <2 bile ducts on MRCP and fiscal ability and availability of daVinci Xi Robot
Chen et. al, 2016	Inclusion criteria were low risk for comorbidity and malignancy, liver/spleen ratio more than 1, graft-to-recipient weight ratio over 0.8%, and remnant liver volume more than 30% of the liver.<br><br>Candidates were at will to choose the robotic procedure after passing the donor evaluation. The selection criteria between the robotic and open procedures were not different during the study period*.

***Selection criteria**

Supplemental Table S4. Donor Complications

Author/Year	Robotic approach (n)	Laparoscopic approach (n)	Open approach (n)
Rho et. al, 2020	- Total complications (12)<br>- Pleural effusion (1)<br>- Hepatic artery bleeding (1)<br>- Others (10)	- Total complications (33)<br>- Biliary stricture (1)<br>- Intestinal obstruction (1)<br>- Others (33)	- Total complications (22)<br>- Biloma (1)<br>- Others (21)
Broering et. al, 2020	- Total complications (2)<br>- Biliary leak (1)<br>- Pulmonary embolism (1)	NA	- Total complications (12)<br>- Biliary leak (2)<br>- Pulmonary embolism (1)<br>- Portal vein stenosis (1)<br>- Ascites (1)<br>- Wound infection (3)<br>- Other (4)
Binoj et. al, 2020	NR	NA	NR
Chen et. al, 2016	- Total complications (1)<br>- Biliary leak (1)	NA	- Total complications (5)<br>- NR

NR: Not Reported, NA: Not Applicable

Supplemental Table S5. Recipients' preoperative and postoperative characteristics

Author/Year	Intervention	Population number	Age (years, mean ±S.D.)	Female (%)	BMI (mean ±S.D.)	MELD scores (n ±S.D.)	Total operative time (min ±S.D.)
**Rho et. al, 2020**	Robotic	52	56.5 (6.6)	21.2	24.3 (3.3)	11.5 (5.1)	493.6 (91.5)
	Laparoscopic	118	53.8 (11.2)	32.2	23.8 (3.6)	16.2 (8.3)	404.4 (47.4)
	Open	62	55.3 (6.7)	20.9	23.9 (3.0)	11.1 (7.4)	355.9 (95.7)
**Broering et. al, 2020**	Robotic	35	59.0 (NR)	42.9	27.1 (7.1)	22.0 (5.2)	504 (73.5)
	Open	70	NR	35.7	NR	NR	331 (65.1)
**Binoj et. al, 2020**	Robotic	51	NR	NR	NR	22.2 (6.2)	536.8 (73.4)
	Open	62	NR	NR	NR	23.9 (6.8)	458.2 (99.1)
**Chen et. al, 2016**	Robotic	13	NR	53.8	NR	NR	596.0 (353.0 – 753.0)*
	Open	54	NR	NR	NR	NR	383.0 (249.0 – 883.0)*
**Total**	Robotic	151	57.5 (0.3)	21.8	25.4 (1.3)	18.1 (4.9)	519.4 (NA)
	Laparoscopic	118	53.8 (11.2)	32.2	23.8 (3.6)	16.2 (8.3)	404.0 (47.4)
	Open	248	55.3 (6.7)	15.3	23.9 (3.0)	17.5 (6.4)	380.3 (NA)

***: range**

Supplemental Table S6. Recipients' transplant indications

Author/Year	Indications (n)
Rho et. al, 2020	Robotic<br>- Hepatocellular carcinoma (28)<br>- End stage liver disease (24)<br><br>Laparoscopic<br>- Hepatocellular carcinoma (45)<br>- End stage liver disease (17)<br><br>Open<br>- Hepatocellular carcinoma (62)<br>- End stage liver disease (56)
Broering et. al, 2020	Robotic<br>- NASH (16)<br>- Viral (10)<br>- Cholestatic (2)<br>- Autoimmune (1)<br>- Other (6)<br><br>Open<br>- NASH (28)<br>- Viral (21)<br>- Cholestatic (7)<br>- Other (14)
Binoj et. al, 2020	NR
Chen et. al, 2016	NR

NR: Not reported, NASH: Non-alcoholic steatohepatitis

Supplemental Table S7. Recipient Complications

Author/Year	Robotic approach (n)	Laparoscopic approach (n)	Open approach (n)
Rho et. al, 2020	- Total complications (24)<br>- Biliary leak (7)<br>- Pulmonary problem (3)<br>- Hepatic artery thrombosis (2)<br>- Portal or hepatic vein problem (1)<br>- Others (11)	- Total complications (70)<br>- Graft subcapsular hematoma (2)<br>- Intra-abdominal bleeding (2)<br>- Intra-abdominal fluid collection (4)<br>- Pulmonary problem (4)<br>- Gastrointestinal problem (3)<br>- Biliary problem (22)<br>- Graft vs host disease (2)<br>- Hepatic artery problem (3)<br>- Portal or Hepatic vein problem (3)	- Total complications (38)<br>- Intra-abdominal fluid collection (2)<br>- Pulmonary problem (2)<br>- Gastrointestinal problem (2)<br>- Renal problem (1)<br>- Biliary problem (23)<br>- Portal or Hepatic vein problem (1)
Broering et. al, 2020	- Total complications (17)<br>- Sepsis (8)<br>- Biliary leak (4)<br>- Bleeding (3)<br>- Hepatic artery thrombosis (2)	NA	- Total complications (37)<br>- Sepsis (12)<br>- Biliary leak (11)<br>- Bleeding (9)<br>- Hepatic artery thrombosis (2)<br>- Portal thrombosis (2)
Binoj et. al, 2020	NR	NA	NR
Chen PD et. al, 2016	- Total complications (2)<br>- Hepatic artery thrombosis (1)<br>- Biliary leak (1)	NA	- Total complications (11)<br>- Hepatic artery thrombosis (2)<br>- Portal vein stenosis (3)<br>- Portal vein thrombosis (2)<br>- Biliary leak (4)

NR: Not Reported, NA: Not Applicable

Supplemental Digital Content Search strategies

<u>Ovid</u>

Database(s): EBM Reviews - Cochrane Central Register of Controlled Trials July 2020, EBM Reviews - Cochrane Database of Systematic Reviews 2005 to August 26, 2020, Embase 1974 to 2020 September 02 , Ovid MEDLINE(R) and Epub Ahead of Print, In-Process & Other Non-Indexed Citations and Daily 1946 to September 02, 2020

Search Strategy:

#	Searches	Results
1	exp Robotic Surgical Procedures/	11223
2	exp Hepatectomy/	87358
3	exp Living Donors/	46931
4	1 and 2 and 3	6
5	exp Liver Transplantation/	175361
6	1 and 5	18
7	(((live or living) and donor* and (robot* or "Intuitive Xi" or "da Vinci" or "minimal invasive surg*" or "minimally invasive surg*" or "minimal invasive procedure*" or "minimally invasive procedure*" or microsurg*) and hepatectom*) or ((robot* or "Intuitive Xi" or "da Vinci" or "minimal invasive surg*" or "minimally invasive surg*" or "minimal invasive procedure*" or "minimally invasive procedure*" or microsurg*) and ((liver or hepatic) adj4 (graft* or retransplant* or transplant*)))).ti,ab,kw.	724
8	4 or 6 or 7	730
9	(exp animals/ or exp nonhuman/) not exp humans/	11163988
10	((alpaca or alpacas or amphibian or amphibians or animal or animals or antelope or armadillo or armadillos or avian or baboon or baboons or beagle or beagles or bee or bees or bird or birds or bison or bovine or buffalo or buffaloes or buffalos or "c elegans" or "Caenorhabditis elegans" or camel or camels or canine or canines or carp or cats or cattle or chick or chicken or chickens or chicks or chimp or chimpanze or chimpanzees or chimps or cow or cows or "D melanogaster" or "dairy calf" or "dairy calves" or deer or dog or dogs or donkey or donkeys or drosophila or "Drosophila melanogaster" or duck or duckling or ducklings or ducks or equid or equids or equine or equines or feline or felines or ferret or	9613324

	ferrets or finch or finches or fish or flatworm or flatworms or fox or foxes or frog or frogs or "fruit flies" or "fruit fly" or "G mellonella" or "Galleria mellonella" or geese or gerbil or gerbils or goat or goats or goose or gorilla or gorillas or hamster or hamsters or hare or hares or heifer or heifers or horse or horses or insect or insects or jellyfish or kangaroo or kangaroos or kitten or kittens or lagomorph or lagomorphs or lamb or lambs or llama or llamas or macaque or macaques or macaw or macaws or marmoset or marmosets or mice or minipig or minipigs or mink or minks or monkey or monkeys or mouse or mule or mules or nematode or nematodes or octopus or octopuses or orangutan or "orang-utan" or orangutans or "orang-utans" or oxen or parrot or parrots or pig or pigeon or pigeons or piglet or piglets or pigs or porcine or primate or primates or quail or rabbit or rabbits or rat or rats or reptile or reptiles or rodent or rodents or ruminant or ruminants or salmon or sheep or shrimp or slug or slugs or swine or tamarin or tamarins or toad or toads or trout or urchin or urchins or vole or voles or waxworm or waxworms or worm or worms or xenopus or "zebra fish" or zebrafish) not (human or humans or patient or patients)).ti,ab,hw,kw.	
11	8 not (9 or 10)	619
12	limit 11 to (editorial or erratum or note or addresses or autobiography or bibliography or biography or blogs or comment or dictionary or directory or interactive tutorial or interview or lectures or legal cases or legislation or news or newspaper article or overall or patient education handout or periodical index or portraits or published erratum or video-audio media or webcasts) [Limit not valid in CCTR,CDSR,Embase,Ovid MEDLINE(R),Ovid MEDLINE(R) Daily Update,Ovid MEDLINE(R) In-Process,Ovid MEDLINE(R) Publisher; records were retained]	16
13	11 not 12	603
14	remove duplicates from 13	413

Scopus

1 TITLE-ABS-KEY(((live or living) and donor* and (robot* or "Intuitive Xi" or "da Vinci" or "minimal invasive surg*" or "minimally invasive surg*" or "minimal invasive procedure*" or "minimally invasive procedure*" or microsurg*) and hepatectom*) OR ((robot* or "Intuitive Xi" or "da Vinci" or "minimal invasive surg*" or "minimally invasive surg*" or "minimal invasive procedure*" or "minimally invasive procedure*" or microsurg*) and ((liver or hepatic) W/4 (graft* or retransplant* or transplant*))))

2 TITLE-ABS-KEY((alpaca OR alpacas OR amphibian OR amphibians OR animal OR animals OR antelope OR armadillo OR armadillos OR avian OR baboon OR baboons OR beagle OR beagles OR bee OR bees OR bird OR birds OR bison OR bovine OR buffalo OR buffaloes OR buffalos OR "c elegans" OR "Caenorhabditis elegans" OR camel OR camels OR canine OR canines OR carp OR cats OR cattle OR chick OR chicken OR chickens OR chicks OR chimp OR chimpanze OR chimpanzees OR chimps OR cow OR cows OR "D melanogaster" OR "dairy calf" OR "dairy calves" OR deer OR dog OR dogs OR donkey OR donkeys OR drosophila OR "Drosophila melanogaster" OR duck OR duckling OR ducklings OR ducks OR equid OR equids OR equine OR equines OR feline OR felines OR ferret OR ferrets OR finch OR finches OR fish OR flatworm OR flatworms OR fox OR foxes OR frog OR frogs OR "fruit flies" OR "fruit fly" OR "G mellonella" OR "Galleria mellonella" OR geese OR gerbil OR gerbils OR goat OR goats OR goose OR gorilla OR gorillas OR hamster OR hamsters OR hare OR hares OR heifer OR heifers OR horse OR horses OR insect OR insects OR jellyfish OR kangaroo OR kangaroos OR kitten OR kittens OR lagomorph OR lagomorphs OR lamb OR lambs OR llama OR llamas OR macaque OR macaques OR macaw OR macaws OR marmoset OR marmosets OR mice OR minipig OR minipigs OR mink OR minks OR monkey OR monkeys OR mouse OR mule OR mules OR nematode OR nematodes OR octopus OR octopuses OR orangutan OR "orang-utan" OR orangutans OR "orang-utans" OR oxen OR parrot OR parrots OR pig OR pigeon OR pigeons OR piglet OR piglets OR pigs OR porcine OR primate OR primates OR quail OR rabbit OR rabbits OR rat OR rats OR reptile OR reptiles OR rodent OR rodents OR ruminant OR ruminants OR salmon OR sheep OR shrimp OR slug OR slugs OR swine OR tamarin OR tamarins OR toad OR toads OR trout OR urchin OR urchins OR vole OR voles OR waxworm OR waxworms OR worm OR worms OR xenopus OR "zebra fish" OR zebrafish) AND NOT (human OR humans or patient or patients))

3 1 and not 2

4 DOCTYPE(ed) OR DOCTYPE(bk) OR DOCTYPE(er) OR DOCTYPE(no) OR DOCTYPE(sh)

5 3 and not 4

6 INDEX(embase) OR INDEX(medline) OR PMID(0* OR 1* OR 2* OR 3* OR 4* OR 5* OR 6* OR 7* OR 8* OR 9*)

7 5 and not 6
